# Excess mortality during the SARS-CoV-2 pandemic in the City of Frankfurt/Main, Germany, in 2020 and 2021, adjusted for age trends and pandemic phases

**DOI:** 10.3205/dgkh000434

**Published:** 2023-04-28

**Authors:** Katrin Steul, Ursel Heudorf, Helmut Uphoff, Bernd Kowall

**Affiliations:** 1 Institute of Occupational, Social and Environmental Medicine, University Medical Centre of the Johannes Gutenberg University, Mainz, Germany; 2 Institute of Hygiene and Environmental Medicine, Justus Liebig University, Giessen, Germany; 3 Department of Infectious Disease Epidemiology, Hessian State Examination and Investigation Office in Health Care (HLPUG), Dillenburg, Germany; 4 Institute for Medical Informatics, Biometry and Epidemiology, University Hospital Essen, Germany

**Keywords:** SARS-CoV-2 pandemic, mortality, population, age-trend, pandemic phases

## Abstract

**Aims:**

Excess mortality during the SARS-CoV-2 pandemic has been studied in many countries. Accounting for population aging has important implications for excess mortality estimates. We show the importance of adjustment for age trends in a small-scale mortality analysis as well as the importance of analysing different pandemic phases for mortality in an urban population.

**Methods:**

Population data for Frankfurt/Main for 2016–2021 were obtained from the Municipal Office of Statistics, City of Frankfurt/Main. Mortality data from 2016 to 2021 were provided by the Hessian State Authority. For standardized mortality ratios (SMR=observed number of deaths divided by the expected number of deaths), the expected number of deaths was calculated in two ways: For SMR_crude_, the mean mortality rate from the years 2016–2019 was multiplied by the total number of residents in 2020 and 2021 separately. For SMR_adjusted_, this procedure was performed separately for five age groups, and the numbers of expected deaths per age group were added.

**Results:**

SMR_crude_ was 1.006 (95% CI: 0.980–1.031) in 2020, and 1.047 (95% CI: 1.021–1.073) in 2021. SMR_adjusted_ was 0.976 (95% CI: 0.951–1.001) in 2020 and 0.998 (95% CI: 0.973–1.023) in 2021. Excess mortality was observed during pandemic wave 2, but not during pandemic waves 1 and 3.

**Conclusion:**

Taking the aging of the population into account, no excess mortality was observed in Frankfurt/Main in 2020 and 2021. Without adjusting for population aging trends in Frankfurt /Main, mortality would have been greatly overestimated.

## Zusammenfassung

**Ziele:**

Die Übersterblichkeit während der SARS-CoV-2-Pandemie wurde in vielen Ländern untersucht. Die Berücksichtigung der Alterung der Bevölkerung hat wichtige Auswirkungen auf die Schätzung der Übersterblichkeit. Wir zeigen, wie wichtig die Berücksichtigung von Alterstrends in einer kleinmaßstäblichen Mortalitätsanalyse ist, und wie wichtig es ist, verschiedene Pandemiephasen für die Mortalität in einer städtischen Bevölkerung zu analysieren.

**Methoden:**

Die Bevölkerungsdaten für Frankfurt am Main für die Jahre 2016 bis 2021 wurden vom Bürgeramt Statistik und Wahlen der Stadt Frankfurt am Main erhalten. Die Mortalitätsdaten 2016 bis 2021 wurden vom Hessischen Landesamt zur Verfügung gestellt. Für die Standardisierte Mortalitätsratio (SMR=beobachtete Zahl der Sterbefälle geteilt durch die erwartete Zahl der Sterbefälle) wurde die erwartete Zahl der Sterbefälle auf zwei Arten berechnet: Für die SMR_roh_ wurde die mittlere Mortalitätsrate aus den Jahren 2016–2019 mit der Gesamtzahl der Bürger im Jahr 2020 bzw. 2021 multipliziert. Für die SMR_adjustiert_ wurde dieses Verfahren für fünf Altersgruppen getrennt durchgeführt und die Zahlen der erwarteten Todesfälle pro Altersgruppe wurden addiert.

**Ergebnisse:**

Die SMR_roh_ betrug 1,006 (95% CI: 0,980–1,031) im Jahr 2020 und 1,047 (95% CI: 1,021–1,073) im Jahr 2021. Die SMR_adjustiert_ betrug 0,976 (95% CI: 0,951–1,001) und 0,998 (95% CI: 0,973–1,023) im Jahr 2021. Eine Übersterblichkeit wurde während der Pandemiewelle 2 beobachtet, nicht jedoch während der Pandemiewellen 1 und 3.

**Schlussfolgerung:**

Unter Berücksichtigung der Alterung der Bevölkerung wurde in den Jahren 2020 und 2021 in Frankfurt am Main keine Übersterblichkeit beobachtet. Ohne Anpassung an die Alterung der Bevölkerung in Frankfurt am Main wäre die Sterblichkeit stark überschätzt worden.

## Introduction

The first cases of SARS-CoV-2 occurred in China by the end of 2019. In Germany, the first case was registered in January 2020. On 1 Febuary 2020, the first returnee with SARS-CoV-2 arrived in Frankfurt/Main via airplane [1]. As of 31 August 2020, 2,665 people with SARS-CoV-2 infections had been reported in Frankfurt, 69 of which were registered as deceased [2]. By the end of 2021 in Frankfurt/Main, 70,825 persons infected with SARS-CoV-2 had been registered, and up to 7,230,304 in Germany [3].

The number of registered cases is strongly influenced by test availability and test regime. Both parameters affect not only the reported number of people infected with SARS-CoV-2, but also the case fatality rate [4], [5], [6], [7], [8], [9]. For the reported cases of death – in contrast to the automated laboratory SARS-CoV-2 reporting system – an additional registration is required. Therefore, under-reporting cannot be ruled out, especially in the case of high volumes of reports. However, the registration data does not distinguish between patients dying *of* or *with* SARS-CoV-2. Accordingly, data on mortality from COVID-19 have only limited informative value. Given limited test availability or a restrictive test regime, the case fatality rate might be overestimated, whereas if registration of deaths with SARS-CoV-2 is incomplete, the mortality of COVID-19 might be underestimated. This limits the assessment of SARS-CoV-2-associated mortality [4], [5], [10], [11]. 

These methodological limitations do not apply when considering all-cause mortality [4], [5], [8], [9], [12], [13], [14]. However, it is not possible to extrapolate mortality from COVID-19 from total mortality. 

Results on excess mortality during the SARS-CoV-2 pandemic vary greatly not only between countries but also within individual countries, depending on the method of analysis [12]. Results on excess mortality strongly depend on whether the aging of the population is taken into account. The importance of age standardization is also evident in analyses by Levitt et al., who estimate an excess mortality of 2.7% for Germany with age standardization for the years 2020 and 2021 combined, and 6.4% without age standardization [12].

These methodological comparisons have so far been published at the country level. This study presents a small-scale analysis of mortality in the City of Frankfurt/Main, Germany. While Frankfurt/Main is considered a “young city” due to marked population growth through birth and immigration, there is also a strong increase in elderly and especially the very old; e.g., the number of 80- to 84-year-olds rose by 24.6% between 2016 and 2020. 

The aim of our study was to investigate excess mortality in the City of Frankfurt/Main during the pandemic (2020 and 2021). Moreover, we examined how strongly the adjustment for age trends in a small-scale mortality analysis affects excess mortality results. Finally, we aimed to analyze excess mortality during the different pandemic phases. Hence, we not only analyzed the pandemic years 2020 and 2021 in total, but also the different pandemic waves and the phases between them.

## Methods

Population data for Frankfurt/Main for 2016–2021 were taken from the annual reports of the Municipal Office of Statistics, City of Frankfurt/Main [15]. The data refer to 31 December of each year. To calculate the SMRs, mid-year populations were used, which were obtained as the average of the population figures of two consecutive years for 31 December. The data on death cases were compiled by the Hessian Statistical Office (HSL) and made available to the state health office (HLPUG), which provided the data to the authors. The data included death reports only of people residing in Frankfurt who had died in the state of Hesse. For each death case, sex, age and date of death was available. Reports of SARS-CoV-2 infections were taken from the RKI (Robert Koch Institute) database [3]. They are available as cases per reporting week and year for different age groups (5-year intervals). The definition of the different waves in Germany was taken from a publication by the RKI: Wave 1 calendar week (CW) 10 to 20/2020; Wave 2 CW 40/2020 to 8/2021; Wave 3 CW 9 to 23/2021; Wave 4 CW 31 to 51/2021 [16]. 

In this study, two kinds of SMRs (standardized mortality ratios) with 95% confidence intervals were estimated: a crude SMR (SMR_crude_) and an age-adjusted SMR (SMR_ad__j__us__t__ed_).

For SMR_crude_, mortality rates for 2016–2019 were calculated by dividing the total number of deaths by the city’s total mid-year population for each year. The mean of the four mortality rates was multiplied by the total city population in 2020 and 2021 separately to give the expected number of deaths for 2020 and 2021. SMR_crude_ was estimated by dividing the observed total number of deaths in Frankfurt for each year, 2020 and 2021, by the expected number of deaths for the corresponding year.

For SMR_adjusted_, mortality rates for 2016–2019 were calculated separately for five age groups (0–29, 30–59, 60–69, 70–79, ≥80 years) for each year. Each age-specific mean mortality rate was multiplied by the population of the corresponding age group in each of the years 2020 and 2021 to give the expected number of deaths for 2020 and 2021 for each age group. The total number of expected deaths for each of the years 2020 and 2021 was obtained by adding the expected numbers of deaths for the five age groups. This total number of expected deaths was the denominator of SMR_adjusted_.

This procedure to estimate SMR_adjusted_ was applied to all single waves and the intermediate phases (summer periods). Instead of whole years, only the calendar weeks of the respective waves (wave 1 to 4) and of the intermediate phases (between waves 1 and 2, and between waves 3 and 4) were taken into account. For wave 2, which lasted from CW 40/2020 to CW 8/2021, age-specific mean mortality rates were calculated by using the respective population on 31 December 2020. 

Finally, weekly SMR_adjusted_ for 2020 and 2021 in the total population and weekly SMRs for persons over 80 years of age were estimated by the procedure described above. In these analyses, age-specific weekly mortality rates for the years 2016 to 2019 were calculated and were used to calculate expected numbers of death for each calendar week. Weekly SMRs were estimated by dividing weekly observed numbers of deaths in each of the years 2020 and 2021 by the weekly expected numbers of death. 

For both ways of calculating the number of expected deaths, we additionally estimated the difference between the observed number of deaths and the expected number of deaths for the years 2020 and 2021.

All analyses were also done separately by sex.

## Results

SMR_crude_ was 1.006 (95% CI: 0.980–1.031) in 2020, and 1.047 (95% CI: 1.021–1.073) in 2021 (Table 1 (Tab. 1)). SMR_crude_ was higher in men than in women in 2020 and in 2021. 

SMR_adjusted_ was 0.976 (95% CI: 0.951–1.001) in 2020, and 0.998 (95% CI: 0.973–1.023) in 2021 (Table 2 (Tab. 2)). SMR_adjusted_ was higher in men than in women in 2020 and 2021. In 2020, the observed number of deaths was 148.5 lower than the expected number of deaths. In 2021, 12.1 fewer deaths were observed than expected.

Table 3 (Tab. 3) shows SMR_adjusted_ for the different pandemic waves and for the intermediate phases during the summer weeks of 2020 and 2021, when incidences of SARS-CoV-2 were low. In waves 1 and 3, the number of observed death cases was –110.5 and –54.6 lower, respectively, than the expected number of death cases (wave 1 SMR_adjusted_ 0.917; 95% CI: 0.866–0.969; wave 3 SMR_adjusted_ 0.970; 95% CI: 0.925–1.015). In waves 2 and 4, the number of observed death cases was 273.9 and 80.4 higher, respectively, than the expected number of deaths (wave 2 SMR_adjusted_ 1.106; 95% CI: 1.066–1.147; wave 4 SMR_adjusted_ 1.033; 95% CI: 0.933–1.073). 

**Figure 1:**
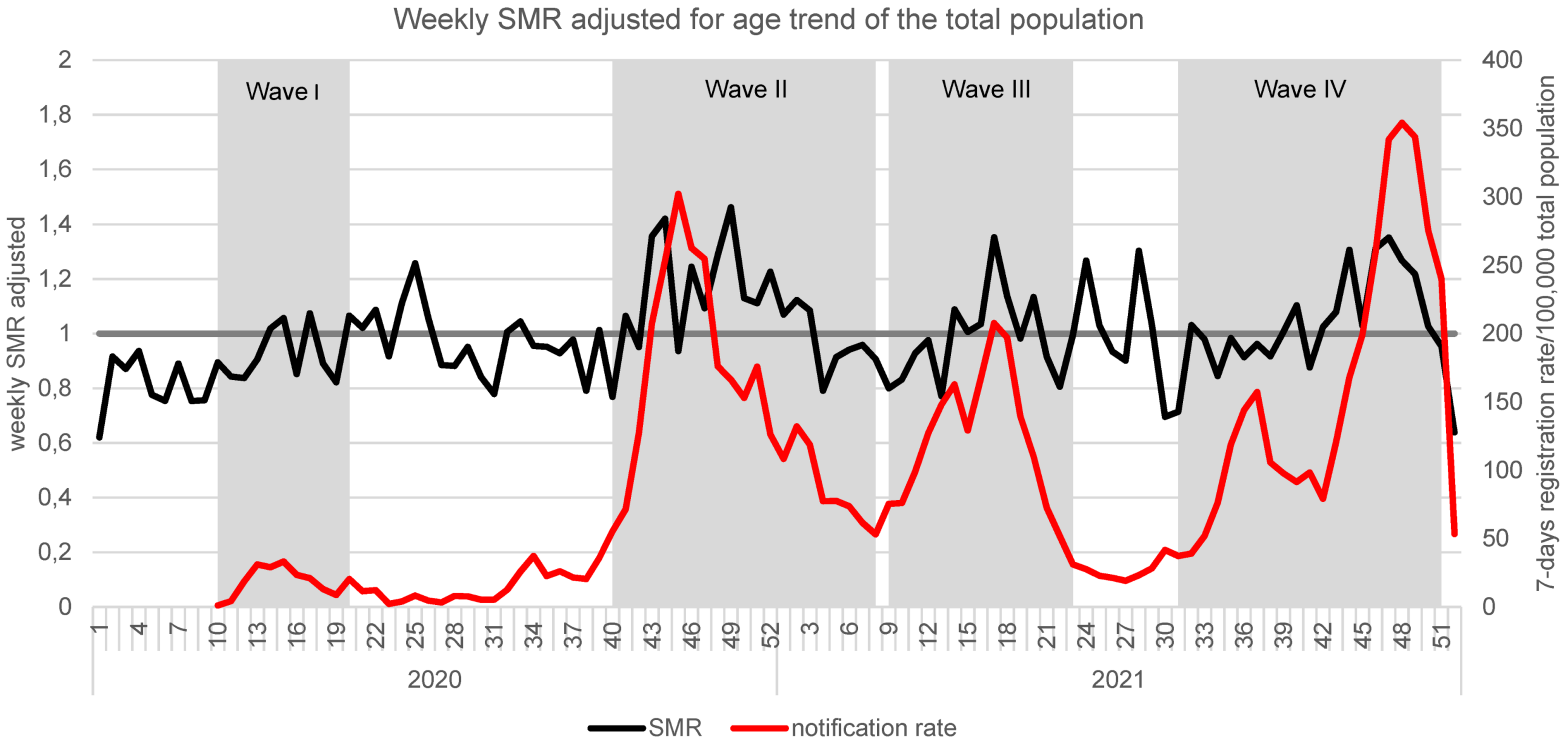
Age-adjusted weekly SMR and respective 7-day registration rates/100,000 in Frankfurt/Main in 2020 and 2021 for the whole population

**Figure 2:**
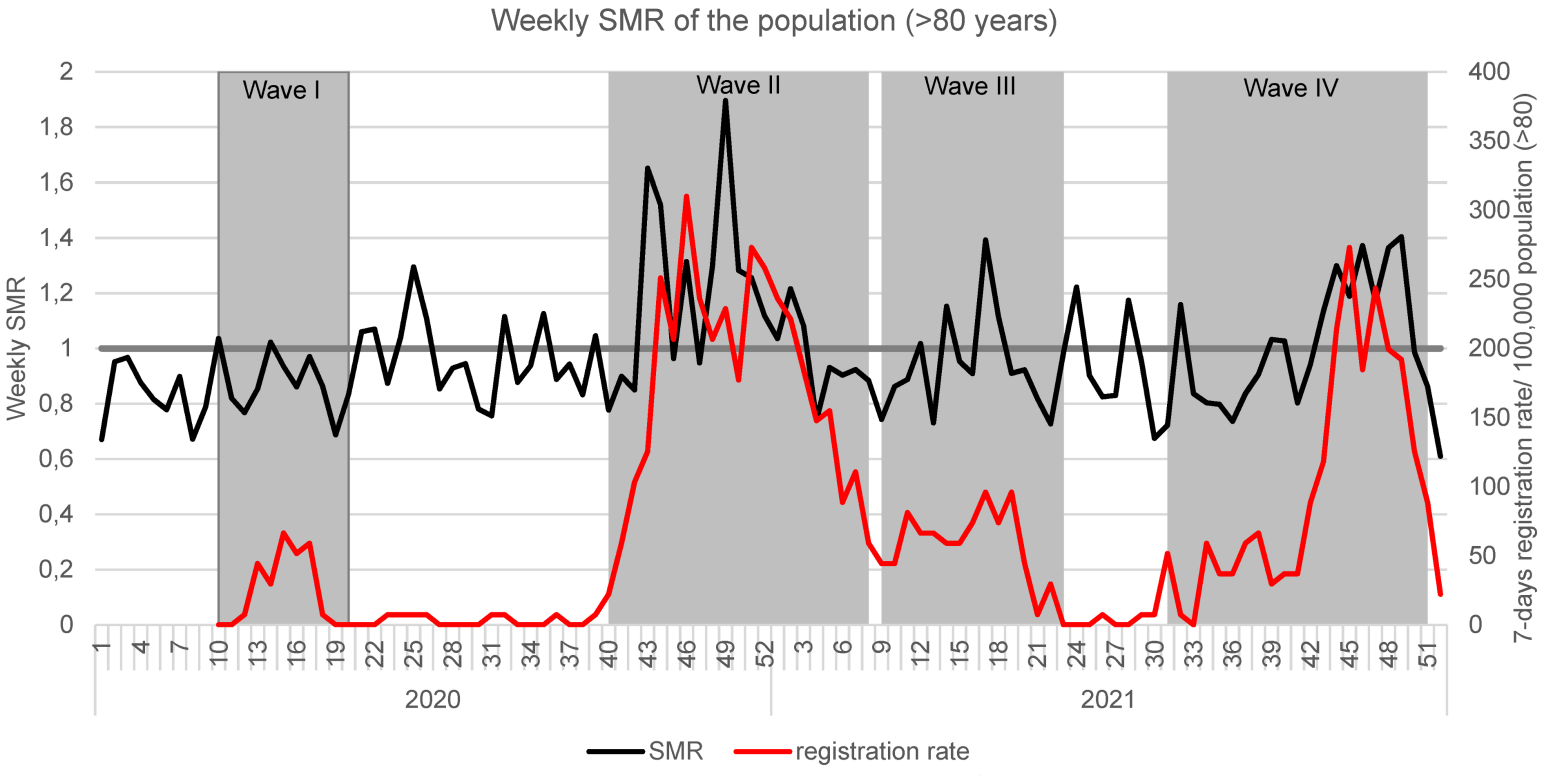
Weekly SMR and respective 7-day registration rates/100,000 in Frankfurt/Main in 2020 and 2021 for the over 80-year-old population

Weekly SMR_adjusted_ for the years 2020 and 2021 in comparison with the years 2016 to 2019 is shown in Figure 1 (Fig. 1) for the total population, and Figure 2 (Fig. 2) exhibits the SMR for the over-80-year-olds. Furthermore, SARS-CoV-2 seven-day reports per 100,000 of the respective population are also shown.

In the total population, registered infections peaked in wave 2 with 302/100,000 in CW 45/2020, in wave 3 with 208/100,000 in calendar week 17/2021 and 354/100,000 in calendar week 48/2021 during wave 4 (Figure 1 (Fig. 1)). Peaks in SMR_adjusted_ during the waves occurred in week 49/2020 with a SMR_adjusted_=1.462 in the second wave, SMR_adjusted_=1.352 in week 17/2021 (3^rd^ wave) and an SMR_adjusted_=1.352 in week 47/2021 (4^th^ wave). 

The highest registered weekly SARS-CoV-2 infection rates in the over 80-year-olds were twice as high as in the general population during the first wave, slightly higher than infection rates among the general population in the second wave at 310/100,000 (CW 46/2020), and lower than those of the general population in the following waves (Figure 2 (Fig. 2)). In wave 2, SMR_adjusted_ reached a maximum of 1.896 in week 49/2020. In 2021, maximum SMR_adjusted_ were 1.393 (3^rd^ wave; calendar week 17/2021) and 1.404 (4^th^ wave, calendar week 49/2021).

## Discussion

No excess mortality (SMR_adjusted_) was observed in Frankfurt/Main in the pandemic years 2020 and 2021 in total, encompassing the circulation of the Wuhan variant as well as the alpha and delta variants in Germany. When analyzing the different pandemic waves separately, excess mortality was seen in wave 2 – autumn/winter of 2020 – but not in waves 1, 3 and 4, in spring of 2020 and 2021, and autumn/winter of 2021, respectively. 

In many countries, significant excess mortality was observed in the first half of 2020. Examples include 102.8% in England and Wales, 99% in Spain, 33.7% in France, 25.1% in Sweden and 25.1% in Italy by the end of August 2020 [11]. In Italy, excess mortalities between +48.8% and maximum 600% were reported in individual provinces and cities during the first wave – with great geographical and temporal heterogeneity [14], [17], [18]. In 2020 – with the first wave and the beginning of the second wave of the SARS-CoV-2 pandemic – excess mortality was somewhat lower in most countries, for example 3% in Sweden, 14.8% in Spain and 8.8% in Switzerland [8], [19]. All publications showed a strong increase in mortality in older age groups and, where analyzed, a higher mortality for men than for women. 

In Germany, excess mortality was comparatively low with less than +1% in the first months of 2020 and until the end of 2020 [20], [21], [22]. Only during a few weeks in the first pandemic wave, excess mortality ranged from 3% to up to 15% – with substantial heterogeneity between federal states as well as between individual counties within these states [9], [21].

Without adjustment for population age development, excess mortality_crude_ in Frankfurt/Main presented within the range to be expected for Germany with +0.6% (SMR_crude_ 1.006 (95% CI 0,980–1.031; +33 deaths) for 2020. However, adjustment for population age trends yielded a significant reduction of –2.4% in mortality_adjusted_. (SMR_adjusted_ 95% CI 0.951–1.001; –148.5 deaths). This change is consistent with the findings of other authors. For instance, Stang et al. reported an excess mortality of +3% (SMR 1.03) in wave 1 in the unadjusted model, with an excess mortality of –2% (SMR 0.98) after adjustment [9]. Gianicolo et al. found excess mortality of +43,835 in the unadjusted model (wave 1, Italy), which decreased to +33,035 after adjusting for population development [23]. The necessity of adjusting for population development was emphasized, especially when considering rapidly aging developed countries such as Germany, where the absolute number of people aged 80 or above increased by approximately 20% from 2016 to 2020. In the absence of adjustment for this development, mortality is necessarily overestimated [13]. The lack of adjustment for population development in many publications as well as in the mortality data published by the WHO in their country comparison was assessed critically [24]. Furthermore, in many countries – especially those with low incomes – there is generally no standardized recording of deaths, which further limits comparability between countries [7]. 

While in most studies only the mortality during the first wave or the combined mortality of the first and second wave were published, we present the mortality for all four pandemic waves until the end of 2021 and separately for the summer phases 2020 and 2021 with low SARS-CoV-2 incidence. In Frankfurt/Main, there was no excess mortality during the first wave – either in the total population or among the residents of the nursing homes in the city [25], [26]. In wave 2, however, there was significant excess mortality_adjusted_ (+10.6%), which particularly affected the over 80-year-olds and, within this group, especially men (+20.7% men, +8.4% women). This correlates with an increase in the incidence of SARS-CoV-2 reporting in the overall population. Many nursing homes for the elderly experienced SARS-CoV-2 outbreaks, including fatalities, despite strict hygiene measures and intense contact restrictions. Rapid SARS-CoV-2 antigen tests were not yet sufficiently available everywhere at that time and vaccines were not available at all [26].

Wave 3 (CW 9–23/2021) was characterized by an increasing spread of the new alpha variant and at the same time by gradual relaxations of the restrictive pandemic measures. At that time, Frankfurt/Main presented an excess mortality_adjusted_ of –3.0%, which was more pronounced among those over 80 years of age (–6.3%), as vaccine rollout in Germany had started at the end of 2020. In accordance with the recommendations of the German Commission on Vaccination (*Ständige Impfkommission*, STIKO), priority was given to vaccinating the very old (>80 years), people at high risk of severe COVID-19 disease and their caregivers, as well as physicians and nursing staff [27]. This needs to be considered when analyzing the change in mortality in this age group.

In the last quarter of 2021, there was another pandemic wave (wave 4) in Germany with SARS-CoV-2 infection rates higher than ever before. At that point, an adjusted excess mortality of +1.5% was recorded for the very old (over 80 years of age) in Frankfurt/Main. Although a large proportion of the older population had already been vaccinated twice by then, the numbers of breakthrough infections led to the STIKO recommending a third vaccination for those older than 70 in October 2021 and for all adults from November 2021 onwards [28], [29]. The potential effects of the vaccinations cannot be quantified precisely in view of other influencing factors (different virus variants, different public health measures, various biases, etc.).

When looking at the weekly Standardized Mortality Ratio (SMR), the excess mortality_adjusted_ in wave 2 is clearly evident (Figure 1 (Fig. 1) and Figure 2 (Fig. 2)), not only in the group of the very old but also in the overall population. In addition, after the end of wave 1, excess mortality_adjusted_ is still noticeable, especially among those older than 80. One possible explanation for this could be a deterioration in the quality of medical care as a result of restrictive measures. In order to discuss this further, information about the quality of medical care in Germany (frequency of check-ups, etc.) during the SARS-CoV-2 pandemic is necessary.

## Limitations and strengths

The Robert Koch Institute’s (RKI) reporting data are subject to various biases, as follows. Test availability and testing regimes have a strong influence on the weekly SARS-CoV-2 reporting rate. Cases of death with or from SARS-CoV-2 depend on the reporting discipline of the physicians confirming the death and the work of the public health authorities. A differentiation between deaths from or with SARS-CoV-2 is not possible without a closer look at each individual case. 

With regard to the proportion of detected virus variants and the definition of the pandemic waves, we refer to the nationwide data of the RKI. We cannot totally exclude possible regional deviations due to a lack of corresponding data. 

Many of the studies published to date include only the first wave [9], [11], [14], [17], [18], the first 6 months of 2020 [21], [22], [23], the period from January to October 2020 [20] or the year 2020 as a whole [8], [13], [19], [30], [31], and therefore consider only phases in which the Wuhan variant was dominant. Only some publications went on to evaluate data up to 2021, when the alpha and delta variants were circulating and vaccine rollout had begun [5], [12], [32], [33], [34], [35], [36], [37]. The strength of our study is that it not only encompasses two complete pandemic years with the circulation of the Wuhan variant of SARS-CoV-2 as well as the alpha and delta variants, but also analyzes the individual pandemic waves and the intermediate phases separately.

## Conclusion

We analyzed data from two full years and four waves of the SARS-CoV-2 pandemic in Frankfurt/Main. The data provide a good overview of overall mortality in Frankfurt/Main, also taking into account the increasing age of the population. There was 0.6% excess mortality_crude_ in 2020 (33 cases) and 4.7% excess mortality in 2021 (+279 cases). Taking into account the trend of increased aging within the population, excess mortality_adjusted_ decreased to –2.4% (–148.5 cases) in 2020 and –0.2% (–12.1 cases) in 2021. This confirms the need for such an adjustment, especially in strongly aging societies.

No excess mortality was observed during the first and third waves of the pandemic. In the second wave, excess mortality adjusted for population development was +10.6% and +3.3% in the fourth wave. The excess mortality rate needs to be considered in relation to complex influencing factors (including virus variants, public health measures, etc.) and cannot be categorically attributed to COVID-19 infection or immunity in the population through previous infection or vaccination. For a more precise analysis of the causes of excess mortality, further analyses with individual data on the exact causes of death and medical histories would be required.

While Germany experienced excess mortality (adjusted for population trends) in 2020 and 2021 [12], the small-area analysis for the city of Frankfurt showed negative excess mortality. This underscores the importance of small-scale analyses for the proper information of the population and as a basis for the planning and control of local public health measures.

## Notes

### Competing interests

The authors declare that they have no competing interests.

## Figures and Tables

**Table 1:**
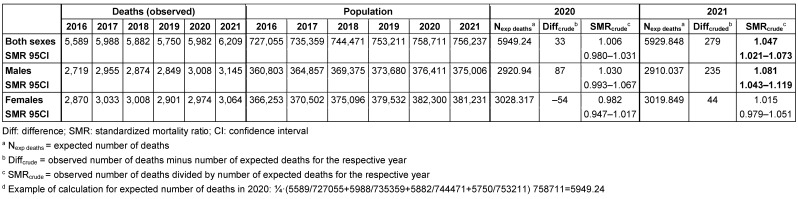
Mortality in Frankfurt/Main, Germany, 2020 and 2021, without adjustment for age trend of the population

**Table 2:**
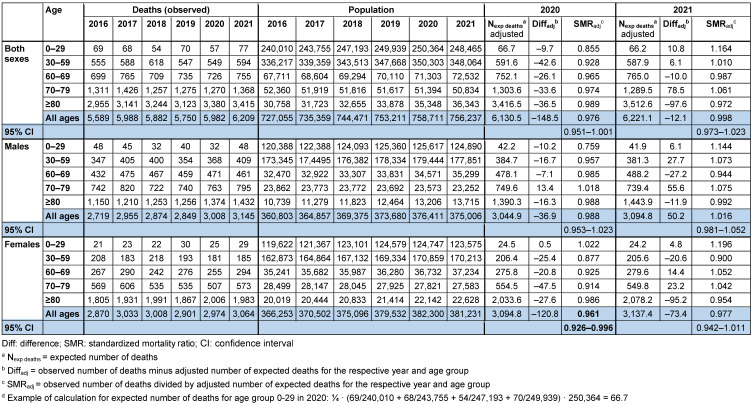
Mortality in Frankfurt/Main, Germany, 2020 and 2021, adjusted for age trend of the population

**Table 3:**
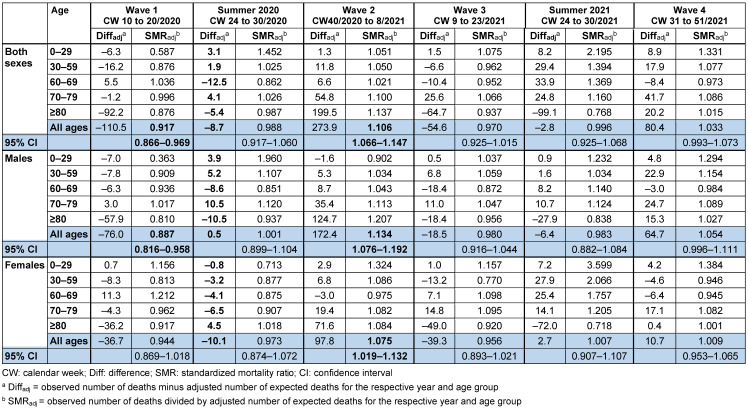
Mortality in Frankfurt/Main, Germany, 2020–2021, adjusted for age trend of the population, during the four pandemic waves and during summer phases

## References

[1] Hoehl S, Rabenau H, Berger A, Kortenbusch M, Cinatl J, Bojkova D, Behrens P, Böddinghaus B, Götsch U, Naujoks F, Neumann P, Schork J, Tiarks-Jungk P, Walczok A, Eickmann M, Vehreschild MJGT, Kann G, Wolf T, Gottschalk R, Ciesek S (2020). Evidence of SARS-CoV-2 Infection in Returning Travelers from Wuhan, China. N Engl J Med.

[2] Heudorf U, Steul K, Gottschalk R (2020). Sars-Cov-2 in children – insights and conclusions from the mandatory reporting data in Frankfurt am Main, Germany, March-July 2020. GMS Hyg Infect Control.

[3] Robert Koch-Institut SurvStat@RKI. Abfrage der Meldedaten nach Infektionsschutzgesetz (IfSG) über das Web.

[4] Beaney T, Clarke JM, Jain V, Golestaneh AK, Lyons G, Salman D, Majeed A (2020). Excess mortality: the gold standard in measuring the impact of COVID-19 worldwide?. J R Soc Med.

[5] COVID-19 Excess Mortality Collaborators (2022). Estimating excess mortality due to the COVID-19 pandemic: a systematic analysis of COVID-19-related mortality, 2020-21. Lancet.

[6] Silveira EA, Noll M, Hallal PC, de Oliveira C (2022). The Need to Use Mortality, and Not Case-Fatality, to Compare COVID-19 Deaths Worldwide. Int J Prev Med.

[7] Helleringer S, Queiroz BL (2022). Commentary: Measuring excess mortality due to the COVID-19 pandemic: progress and persistent challenges. Int J Epidemiol.

[8] Locatelli I, Rousson V (2021). A first analysis of excess mortality in Switzerland in 2020. PLoS One.

[9] Stang A, Standl F, Kowall B, Brune B, Böttcher J, Brinkmann M, Dittmer U, Jöckel KH (2020). Excess mortality due to COVID-19 in Germany. J Infect.

[10] Kelly G, Petti S, Noah N (2021). Covid-19, non-Covid-19 and excess mortality rates not comparable across countries. Epidemiol Infect.

[11] Achilleos S, Quattrocchi A, Gabel J, Heraclides A, Kolokotroni O, Constantinou C, Pagola Ugarte M, Nicolaou N, Rodriguez-Llanes JM, Bennett CM, Bogatyreva E, Schernhammer E, Zimmermann C, Costa AJL, Lobato JCP, Fernandes NM, Semedo-Aguiar AP, Jaramillo Ramirez GI, Martin Garzon OD, Mortensen LH, Critchley JA, Goldsmith LP, Denissov G, Rüütel K, Le Meur N, Kandelaki L, Tsiklauri S, O’Donnell J, Oza A, Kaufman Z, Zucker I, Ambrosio G, Stracci F, Hagen TP, Erzen I, Klepac P, Arcos González P, Fernández Camporro Á, Burström B, Pidmurniak N, Verstiuk O, Huang Q, Mehta NK, Polemitis A, Charalambous A, Demetriou CA (2022). Excess all-cause mortality and COVID-19-related mortality: a temporal analysis in 22 countries, from January until August 2020. Int J Epidemiol.

[12] Levitt M, Zonta F, Ioannidis JPA (2022). Comparison of pandemic excess mortality in 2020-2021 across different empirical calculations. Environ Res.

[13] De Nicola G, Kauermann G, Höhle M (2022). On assessing excess mortality in Germany during the COVID-19 pandemic. AStA Wirtsch Sozialstat Arch.

[14] Odone A, Delmonte D, Gaetti G, Signorelli C (2021). Doubled mortality rate during the COVID-19 pandemic in Italy: quantifying what is not captured by surveillance. Public Health.

[15] Stadt Frankfurt am Main Statistisches Jahrbuch Frankfurt am Main.

[16] Schilling J, Buda S, Tolksdorf K (2022). Zweite Aktualisierung der „Retrospektiven Phaseneinteilung der COVID-19-Pandemie in Deutschland“. Epid Bull.

[17] Scortichini M, Schneider Dos Santos R, De’ Donato F, De Sario M, Michelozzi P, Davoli M, Masselot P, Sera F, Gasparrini A (2021). Excess mortality during the COVID-19 outbreak in Italy: a two-stage interrupted time-series analysis. Int J Epidemiol.

[18] Sandrini M, Andreano A, Murtas R, Tunesi S, Riussi A, Guido D, Greco MT, Gattoni ME, Gervasi F, Consolazio D, Adreoni L, Decarli A, Russo AG (2020). Assessment of the Overall Mortality during the COVID-19 Outbreak in the Provinces of Milan and Lodi (Lombardy Region, Northern Italy). Epidemiol Prev.

[19] Kowall B, Standl F, Oesterling F, Brune B, Brinkmann M, Dudda M, Pflaumer P, Jöckel KH, Stang A (2021). Excess mortality due to Covid-19? A comparison of total mortality in 2020 with total mortality in 2016 to 2019 in Germany, Sweden and Spain. PLoS One.

[20] Morfeld P, Timmermann B, Groß VJ, Lewis P, Erren TC (2021). COVID-19: Wie änderte sich die Sterblichkeit? – Mortalität von Frauen und Männern in Deutschland und seinen Bundesländern bis Oktober 2020. Dtsch Med Wochenschr.

[21] Morfeld P, Timmermann B, Groß JV, Lewis P, Erren TC (2021). Vor, während und nach der ersten COVID-19-Welle: Sterblichkeitsanalysen zeigen relevante Trends in Deutschland und seinen Bundesländern bis Juni 2020. Gesundheitswesen.

[22] Morfeld P, Timmermann B, Groß JV, Lewis P, Cocco P, Erren TC (2021). COVID-19: Heterogeneous Excess Mortality and “Burden of Disease” in Germany and Italy and Their States and Regions, January-June 2020. Front Public Health.

[23] Gianicolo EAL, Russo A, Büchler B, Taylor K, Stang A, Blettner M (2021). Gender specific excess mortality in Italy during the COVID-19 pandemic accounting for age. Eur J Epidemiol.

[24] Van Noorden R (2022). COVID death tolls: scientists acknowledge errors in WHO estimates. Nature.

[25] Heudorf U, Müller M, Schmehl C, Gasteyer S, Steul K (2020). COVID-19 in long-term care facilities in Frankfurt am Main, Germany: incidence, case reports, and lessons learned. GMS Hyg Infect Control.

[26] Heudorf U, Gottschalk R, Müller M, Steul KS (2022). Die SARS-CoV-2-Pandemie in Altenpflegeheimen: Erkenntnisse und Analysen in Frankfurt am Main von März 2020 bis September 2021. Gesundheitswesen.

[27] Vygen-Bonnet S, Koch J, Bogdan C, Harder T, Heininger U, Kling K, Littmann M, Meerpohl J, Meyer H, Schmid-Küpke N, Scholz S, Terhardt M, Treskova-Schwarzbach M, Überla K, van der Sande M, Wichmann O, Wicker S, Wiedermann U, Wild V, von Kries R (2021). Beschluss und Wissenschaftliche Begründung der Ständigen Impfkommission (STIKO) für die COVID-19-Impfempfehlung. Epid Bull.

[28] Ständige Impfkommission (2021). Beschluss der STIKO zur 12. Aktualisierung der COVID-19-Impfempfehlung. Epid Bull.

[29] Koch J, Vygen-Bonnet S, Harder T, Ledig T, Mertens T, Michaelis K, Schönfeld V, Schmid-Küpke N, Steffen A, Wichmann O, Wicker S, Überla K (2021). STIKO-Empfehlung zur COVID-19-Auffrischimpfung mit einem mRNA-Impfstoff für Personen ≥ 70 Jahre und bestimmte Indikationsgruppen sowie Empfehlung zur Optimierung der Grundimmunisierung mit einem mRNA-Impfstoff nach vorausgegangener Impfung mit der COVID-19 Vaccine Janssen und die dazugehörige wissenschaftliche Begründung. Epid Bull.

[30] Robert Koch-Institut Coronavirus Disease 2019 (COVID-19): Daily Situation Report 30/10/2020.

[31] Onozuka D, Tanoue Y, Nomura S, Kawashima T, Yoneoka D, Eguchi A, Ng CFS, Matsuura K, Shi S, Makiyama K, Uryu S, Kawamura Y, Takayanagi S, Gilmour S, Hayashi TI, Miyata H, Sera F, Sunagawa T, Takahashi T, Tsuchihashi Y, Kobayashi Y, Arima Y, Kanou K, Suzuki M, Hashizume M (2022). Reduced mortality during the COVID-19 outbreak in Japan, 2020: a two-stage interrupted time-series design. Int J Epidemiol.

[32] Wollschläger D, Schmidtmann I, Fückel S, Blettner M, Gianicolo E (2022). Erklärbarkeit der altersadjustierten Übersterblichkeit mit den COVID-19-attribuierten Sterbefällen von Januar 2020 bis Juli 2021. Bundesgesundheitsblatt Gesundheitsforschung Gesundheitsschutz.

[33] Wollschläger D, Gianicolo E, Blettner M, Hamann R, Herm-Stapelberg N, Schoeps M (2021). Association of COVID-19 mortality with COVID-19 vaccination rates in Rhineland-Palatinate (Germany) from calendar week 1 to 20 in the year 2021: a registry-based analysis. Eur J Epidemiol.

[34] Nomura S, Eguchi A, Tanoue Y, Yoneoka D, Kawashima T, Suzuki M, Hashizume M (2022). Excess deaths from COVID-19 in Japan and 47 prefectures from January through June 2021. Public Health.

[35] Manz KM, Batcha AMN, Mansmann U (2022). Regionale und zeitliche Trends der SARS-CoV-2 assoziierten Sterblichkeit in Bayern: Eine altersstratifizierte Analyse über 5 Quartale für Personen ab 50 Jahren. Gesundheitswesen.

[36] Manz KM, Schwettmann L, Mansmann U, Maier W (2022). Area Deprivation and COVID-19 Incidence and Mortality in Bavaria, Germany: A Bayesian Geographical Analysis. Front Public Health.

[37] Locatelli I, Rousson V (2022). Mortality in Switzerland in 2021. PLoS One.

